# Restoration of the Korringa Relation in Disordered Liquid Systems via Transverse Relaxation (T_2_)

**DOI:** 10.3390/ma19091826

**Published:** 2026-04-29

**Authors:** Yuan Zeng, Lanlan Yang, Jiejun Yao, Wei Tang, Xiaolong Liu

**Affiliations:** School of Materials, Sun Yat-sen University, Shenzhen 518107, China; zengy253@mail2.sysu.edu.cn (Y.Z.);

**Keywords:** liquid metals, knight shift, korringa relation, spin-lattice relaxation, spin-spin relaxation

## Abstract

This study resolves the apparent breakdown of the Korringa relation in disordered liquid metals by investigating Ga-based alloys (EGaIn and Galinstan). By integrating temperature-dependent Knight shifts (K) with longitudinal (T_1_) and transverse (T_2_) relaxation measurements, we demonstrate that deviations from classical behavior arise from neglecting transverse spin dephasing induced by structural and electronic disorder. While solid-state alloys follow the conventional Korringa law, the liquid phase exhibits significant discrepancies between T_1_ and T_2_ due to enhanced electron scattering and fluctuating hyperfine fields. By explicitly incorporating T_2_ into a modified framework, the proportionality between the Knight shift and nuclear relaxation is quantitatively restored. This establishes transverse relaxation as a critical parameter for describing nuclear spin dynamics in complex liquid metals, reinforcing NMR as a powerful local probe for optimizing next-generation liquid metal technologies.

## 1. Introduction

Liquid metals (LMs) are a unique class of materials that exhibit both metallic and fluidic characteristics. They have found broad applications in various fields, including electronics, mechanical engineering, biomedical applications, and energy [1,2,3,4,5,6,7,8,9,10]. While several pure metals, such as mercury (−39 °C), cesium (28.4 °C), gallium (29.8 °C), and rubidium (38.89 °C), are liquid at low temperatures, only gallium is widely applicable due to the toxicity or high chemical reactivity of the others. Gallium-based liquid metals exhibit excellent thermal and electrical conductivities, along with low viscosity and low toxicity [11]. Gallium readily alloys with most metals and can form low-melting-point alloys with elements such as indium, bismuth, tin, lead, zinc, and aluminum [12]. With melting points at or below room temperature, Ga-based liquid metals offer flexibility, stretchability, and reconfigurability, enabling multifunctional devices such as actuators, flexible circuits, biomedical devices, and self-healing superconductors [13,14,15]. The development of compact, high-performance X-ray sources is critical for advancements in medical imaging, materials science, and semiconductor metrology. Liquid metals, notably gallium-based alloys, provide unique benefits over traditional solid targets. Ga-In and Ga-In-Sn systems exhibit room-temperature liquidity, high atomic numbers, and minimal vapor pressure, allowing for high-flux, self-refreshing X-ray emission platforms. Gallium-based liquid metal alloys, particularly Ga-In (gallium-indium) and Ga-In-Sn (gallium-indium-tin), are emerging as promising candidates for compact, high-brightness X-ray sources [16]. Their unique combination of low melting points, high thermal conductivities, tunable emission spectra, and self-healing surfaces makes them well-suited for advanced X-ray generation techniques, including microfocus tubes and laser plasma-based systems [17]. These alloys produce multi-line emission spectra due to the simultaneous presence of Ga, In, and Sn, useful for soft and medium X-ray imaging, X-ray fluorescence (XRF) excitation of a wide range of elements, and phase-contrast imaging. These alloys are non-toxic, non-volatile, and possess excellent wetting behavior on various materials, facilitating integration into microfluidic jet systems or static pools. Their low vapor pressure allows operation in a vacuum without substantial metal vapor deposition. Liquid metal targets eliminate pitting and degradation seen in solid targets. Ga-In and Ga-In-Sn provide a self-healing target surface under high power densities. High-pressure jets provide a continuously renewed target, increasing lifespan and efficiency. Combined with femtosecond lasers, they enable the generation of ultrashort pulses of hard/soft X-rays. High thermal conductivity facilitates effective heat dissipation during continuous operation. Ga-In and Ga-In-Sn liquid metal alloys represent a versatile and powerful class of materials for advanced X-ray generation.

In EUV lithography, gallium (Ga), indium (In), and tin (Sn) have been investigated as liquid metal targets for laser-produced plasma (LPP) sources due to their relatively low melting points and strong line emission in the 10–20 nm spectral range. Among them, tin (Sn) is the industry standard, as it provides efficient EUV emission at 13.5 nm, the optimal wavelength for high-resolution photolithography, via 4d–4f and 4d–5p transitions from highly charged Sn ions (Sn^8+^-Sn^14+^). The generation of LPP in liquid metals is critically influenced by short-range atomic structure and the local electronic environment. Traditional characterization methods, such as X-ray diffraction (XRD), lack the spatial and electronic sensitivity required to probe the dynamic, disordered structures and local electron density variations inherent to the liquid phase. To address these limitations, solid-state Nuclear Magnetic Resonance (NMR) serves as a vital complementary approach; by measuring relaxation times, NMR provides a localized probe of electronic states, conduction electron density, and electron-nucleus coupling strength. Central to this analysis is the Korringa relation, a fundamental principle in condensed matter physics that establishes a quantitative link between two key experimental observables: the Knight shift (K), representing static electronic properties, and the nuclear spin-lattice relaxation rate (1/T_1_), representing dynamic fluctuations. The classical Korringa relation, is derived under the assumptions of a homogeneous electron gas, isotropic hyperfine coupling, and well-defined quasiparticle lifetimes. These assumptions are often violated in liquid metals and alloys. Does the apparent ‘breakdown’ of the Korringa relation in disordered liquid alloys represent a fundamental failure of the theory? The disorder refers to liquid metallic systems that lack long-range crystallographic order but retain short-range atomic correlations, resulting in spatial and temporal fluctuations in the local electronic structure and hyperfine fields. We now explicitly distinguish this behavior from that of solid metals or alloys described by a homogeneous electron gas model. In this work, we demonstrate that this breakdown is not intrinsic, but rather arises from neglecting transverse spin dephasing (T_2_) effects induced by structural and electronic disorder. By explicitly incorporating T_2_ into the analysis of nuclear spin relaxation, we show that the Korringa relationship can be quantitatively restored in disordered liquid metallic systems.

## 2. Materials and Characterization

### 2.1. Materials

These materials, including Ga66In20.5Sn13.5 (Galinstan, melting point: 8 °C) and Ga75In25 (EGaIn, melting point: 16 °C), were obtained from Dongguan Huatai Metal Materials Technology Co., Ltd. (Dongguan, China).

### 2.2. Characterization

All solid-state NMR spectra were measured at 9.4 T using a Bruker Avance Av 400 MHz NMR spectrometer (Bruker BioSpin GmbH, Rheinstetten, Germany) with a double resonance 4-mm probe head. A 1 M aqueous solution of Ga(NO_3_)_3_ is used as the primary chemical shift reference for ^71^Ga NMR, with its resonance assigned to 0 ppm. A 0.1 M aqueous solution of In(NO_3_)_3_ is used as the primary chemical shift reference for ^115^In NMR, with its resonance set to 0 ppm. For the ^71^Ga and ^115^In experiment, the 90 ° pulse length is 4.5 μs, and the recycling delay was 1s. It is necessary to wait for 40 min to ensure thermal equilibrium before performing spin-lattice relaxation (T_1_) measurements.

## 3. Results and Discussion

**Figure 1:**
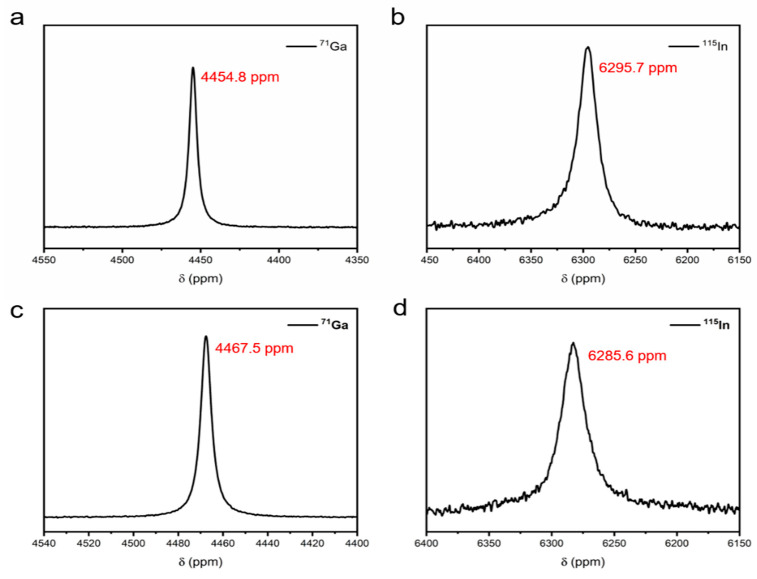
(**a**) ^71^Ga and (**b**) ^115^In NMR spectra of EGaIn; (**c**) ^71^Ga and (**d**) ^115^In NMR spectra of Galinstan at room temperature (300 K).

These materials, including Ga_66_In_20.5_Sn_13.5_ (Galinstan, melting point: 8 °C) and Ga_75_In_25_ (EGaIn, melting point: 16 °C), were selected as model systems for investigation. Figure 1 presents the ^71^Ga and ^115^In NMR spectra of EGaIn and Galinstan. The chemical shift difference highlights the compositional effect of tin by comparing Galinstan, the eutectic alloy of gallium, indium, and tin, with EGaIn, a binary eutectic alloy of gallium and indium. Compared to ^115^In, ^71^Ga was chosen for its smaller quadrupole moment and consequently narrower lines and reduced quadrupolar broadening, which make it more suitable for probing site symmetry, bonding environments, and atomic mixing in multicomponent liquid metals.

**Figure 2:**
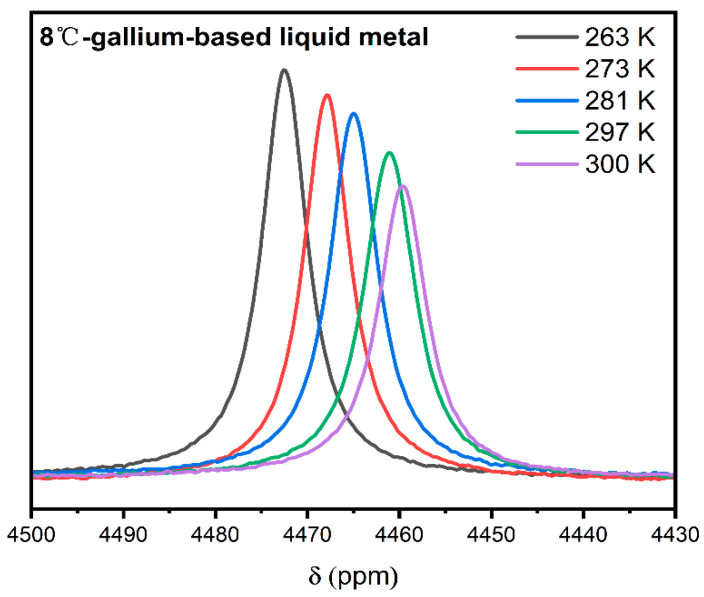
^71^Ga NMR spectra of 8 °C-gallium-based liquid metal (Galinstan) at different temperatures.

**Figure 3:**
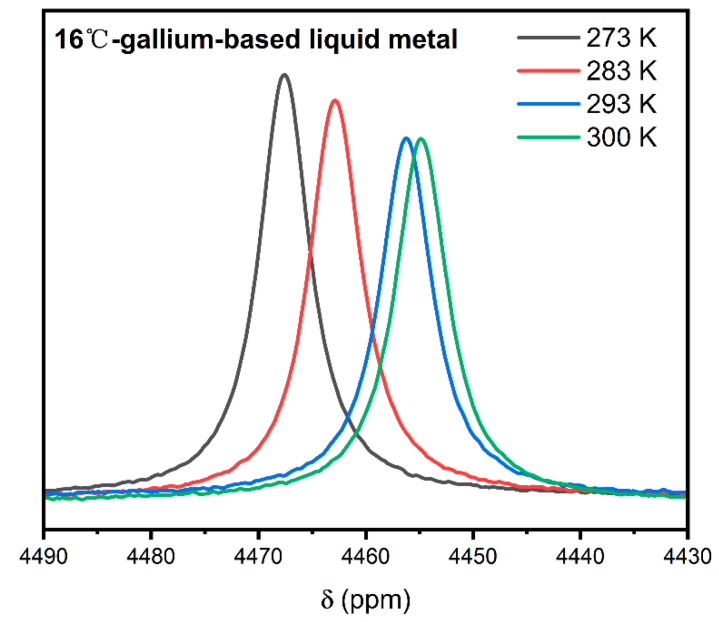
^71^Ga NMR spectra of 16 °C-gallium-based liquid metal (EGaIn) at different temperatures.

Figure 2 and Figure 3 display the ^71^Ga NMR spectra of EGaIn and Galinstan at various temperatures, with corresponding extended data provided in Figures S1 and S2, respectively. The total measured resonance shift, δ_obs_, is composed of two distinct contributions: the Knight shift (K), arising from the hyperfine interaction with conduction electrons, and the chemical shift (δ_cs_), which reflects the local orbital shielding environment. The linewidth is governed by the distribution of these shifts across the sample, encompassing variations in both chemical environments and local carrier concentrations. At elevated temperatures, the observed reduced intensity of the NMR signal can be attributed to an increase in inhomogeneity in the Knight shift, likely resulting from enhanced carrier mobility and thermally activated redistribution of charge carriers, which lead to a wider distribution of local hyperfine fields experienced by the nuclei [18].

In metals, the Knight shift dominates the NMR shift, a result of hyperfine interactions between conduction electrons and the nucleus. The origin of hyperfine interactions and their relevance to nuclear magnetic resonance (NMR) in metals have been thoroughly discussed in standard texts [19,20], as well as in specialized review articles on NMR in solid metals [21]. While the specific functional equations differ between semiconductors and liquid metals, the fundamental physical principle remains consistent: relaxation is governed by the density and behavior of electronic states at the Fermi level. The magnitude and sign of the Knight shift offer insights into metallic bonding and conduction behavior. The Knight shift is a fundamental parameter in nuclear magnetic resonance (NMR) spectroscopy that arises from the interaction between conduction electrons and nuclear spins in metallic or semiconducting systems [22,23]. First observed by W.D. Knight in 1949, this shift reflects the hyperfine coupling between the nuclear magnetic moment and the spin density of conduction electrons at the nucleus [24]. The NMR Knight shift depends upon the conduction electron density at the nucleus under observation. Their NMR shifts are sensitive to temperature variations, indicating that the shift is temperature-dependent. This reflects changes in the electronic density of states at the Fermi level and variations in the electron-nucleus hyperfine interaction strength. Such behavior offers important insights into the continuous temperature-dependent modification of local electronic structure in both liquid and solid phases.

Spin-lattice relaxation time (T_1_) measurements of ^71^Ga in the liquid alloys Galinstan and EGaIn, shown in Figures S3 and S4, were performed to probe their dynamic behavior and electronic structure. T_1_ measurements illustrate the standard inversion-recovery fitting procedure and reveal the strength of electron-nucleus interaction, offering insights into conduction electron density and scattering dynamics. In metallic systems, nuclear spin-lattice relaxation is primarily governed by interactions between nuclear spins and conduction electrons. This relaxation mechanism is often described by the Korringa relation, which establishes a quantitative link between the nuclear spin-lattice relaxation rate (1/T_1_) and the Knight shift (K) in metals. It applies to systems where conduction electrons behave as non-interacting (independent) particles, such as in simple metals or nearly free electron systems. The Korringa relation [20,25,26] is

$$T_{1}TK^{2}=\frac{T}{R_{1}}K^{2}=\frac{\hbar }{4\pi k_{B}}\frac{{\gamma_{e}}^{2}}{{\gamma_{n}}^{2}}$$

where T_1_ is the spin-lattice relaxation time of the nucleus; T is the absolute temperature; K is the Knight shift, the shift in resonance frequency of the nucleus due to hyperfine coupling with conduction electrons; γ_e_ is the gyromagnetic ratio of the electron (γe = −1.76085962784(55) × 1011 s − 1⋅T − 1); γn is the gyromagnetic ratio of the nucleus (γ(71Ga) = 8.1731 × 107 s − 1⋅T − 1) [27]; ℏ is the reduced Planck constant; kB is the Boltzmann constant. Then

$$T_{1}TK^{2}=28.2\times 10^{-7}s\cdot K$$

Table 1 and Table 2 summarize the T_1_ relaxation times of two gallium-based liquid metals across various temperatures.

By applying the Korringa relation, the mean Knight shifts for solid-state EGaIn and Galinstan were calculated. The determination of the sign of the Knight shift induced by electrons or holes is a nontrivial matter, as it depends intricately on both the microscopic mechanism of the hyperfine interaction, such as Fermi contact, dipolar, core polarization, or orbital contributions, and the character of the charge carriers (i.e., their spin and orbital composition near the Fermi level). This complexity is particularly relevant in semimetals, transition metals, and even in certain liquid metals such as liquid gallium [26]. In the case of ^71^Ga, which is commonly studied in both solid and liquid states, the Fermi contact interaction from delocalized s-like conduction electrons dominates the Knight shift; therefore, the sign of the Knight shift is positive. This is consistent with its behavior in the metallic phase, where the hyperfine field generated at the nuclear site by the spin polarization of conduction electrons aligns parallel to the external magnetic field.

**Figure 4:**
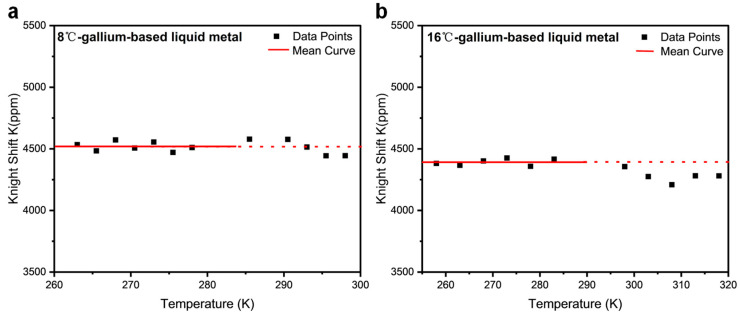
^71^Ga Knight shift versus temperature (T) scatter plot for (**a**) Galinstan (263–298 K) and (**b**) EGaIn (258–318 K).

Figure 4 presents the Knight shift (K) versus temperature (T) scatter plots for EGaIn and Galinstan alloys. The mean Knight shifts for solid-state EGaIn and Galinstan were calculated to be 4390 ± 40 ppm and 4520 ± 50 ppm in the solid-state regime, respectively. The position of the NMR reflects contributions from both the chemical shift, arising from the local electronic environment, and the Knight shift, which originates from hyperfine interactions with mobile charge carriers. Compared with the resonance shifts in Figures S1 and S2, the Knight shift is the primary contributor in these metallic systems, while the chemical shift remains negligible by comparison. Both systems exhibit a weak temperature dependence of the Knight shift in the solid state, consistent with metallic behavior. However, as the temperature increases into the liquid phase, a slight downward trend emerges. This deviation from ideal Fermi-liquid behavior may be attributed to local structural fluctuations in the liquid state. The Knight shift in alloys in the solid state shows weak temperature dependence and follows the Korringa relation reasonably well, consistent with a metallic electronic structure and Fermi-liquid-like behavior of the conduction electrons. Metallic bonding arises from the delocalization of valence electrons, which are not bound to individual atoms but instead form a collective electron cloud permeating the lattice of positively charged atomic cores. This concept is encapsulated in the classical Drude-Lorentz free-electron model, wherein conduction electrons are modeled as non-interacting particles moving freely within a uniform positive background potential formed by the ion cores [28]. In metallic systems, the Korringa relation is kept, and the Knight shift provides a direct probe of the local electronic environment, particularly the density of states at the Fermi level. It manifests as a temperature-independent shift in the NMR frequency of the nucleus, which contrasts with the chemical shift that arises from localized orbital electrons and is typically sensitive to molecular structure and bonding. However, in the liquid metallic state, the mobility of atomic cores introduces dynamic disorder, adding significant complexity to the electronic structure and making the prediction of charge distribution more challenging. Electron-electron interactions enhance the nuclear spin-lattice relaxation rate to a greater extent than the Knight shift, leading to a breakdown of the proportionality predicted by the Korringa relation [29,30]. This dynamic environment leads to notable deviations from classical Drude transport behavior, including enhanced momentum relaxation and modifications to the electronic density of states (DOS) near the Fermi level [31,32]. These effects reflect the breakdown of the free-electron approximation in the liquid metallic state and highlight the importance of electron-ion and electron-electron interactions in determining their transport and spectral properties. When momentum relaxation is strong, conduction electrons undergo frequent scattering events, which rapidly randomize their velocities and suppress coherent propagation. This enhanced scattering increases the temporal fluctuations of the local magnetic field at the nucleus, thereby accelerating nuclear spin-lattice relaxation and resulting in a shorter spin-lattice relaxation time T_1_ [20]. In our measurements (Table 1 and Table 2), we observe a clearly short spin-lattice relaxation time (T_1_) at the liquid metallic state, consistent with strong momentum relaxation of conduction electrons. This indicates enhanced electron scattering processes that generate rapid temporal fluctuations in the local hyperfine field at the nucleus, effectively accelerating nuclear relaxation dynamics.

In a liquid metal at high temperatures, the atoms move so quickly that the local magnetic fields fluctuate on a timescale much shorter than the Larmor precession period. This “averages out” the local field inhomogeneities very efficiently. In the extreme narrowing limit (ω_0_τ_c_ « 1, (τ_c_, the correlation time, i.e., the time between molecular collisions or jumps; ω_0_, the Larmor frequency), which is characteristic of low-viscosity liquids, the rapid motion of atoms results in the longitudinal (spin-lattice) and transverse (spin-spin) relaxation times being equal (T_1_ = T_2_) [33,34].

T_2_ = (πΔυ)^−1^

Δυ is the full width at half maximum (FWHM) of the NMR line. However, the deviations between T_1_ and T_2_ in Ga-based alloys are clearly shown in Table 1 and Table 2. The deviations between T_1_ and T_2_ in Ga-based alloys suggest that gallium atoms have formed short-range clusters with indium atoms or remnants of the solid-state covalent bonding found in α-gallium. Thermal agitation cannot effectively overcome the residual short-range order of the gallium-indium clusters, resulting in a highly disordered, homogeneous melt where the extreme narrowing limit is strictly satisfied. In an ideal liquid metal, the electrons are treated as a “free electron gas” with a uniform (N(E_F_)). Liquid alloys clearly demonstrate a departure from simple Korringa behavior. The divergence from the theoretical Korringa constant suggests that the transition to an ideal metallic liquid is hindered by persistent correlated electronic-nuclear states.

The classical Korringa relation is derived under the assumption of a homogeneous electron gas and isotropic hyperfine coupling, where nuclear spin relaxation is driven solely by fluctuations at the Larmor frequency. Under these ideal conditions, the longitudinal (T_1_) and transverse (T_2_) relaxation times are expected to be equal. Therefore, applying the Korringa law independently to T_2_ lacks rigorous theoretical justification in its original, formal derivation. However, in disordered liquid metals, T_1_ frequently underestimates the total electronic scattering rate because it fails to account for low-frequency or quasi-static fluctuations. In contrast, T_2_ captures these additional dephasing channels, such as structural disorder and electronic scattering, which are directly relevant to the formation of the Knight shift in complex liquids. Consequently, utilizing T_2_ should not be framed as a conventional application of the Korringa law, but rather as a physically motivated generalization. This modified relationship is intended to restore the proportionality between the Knight shift and nuclear relaxation by accounting for the full spectrum of electronic spin fluctuations inherent in disordered systems.

**Figure 5:**
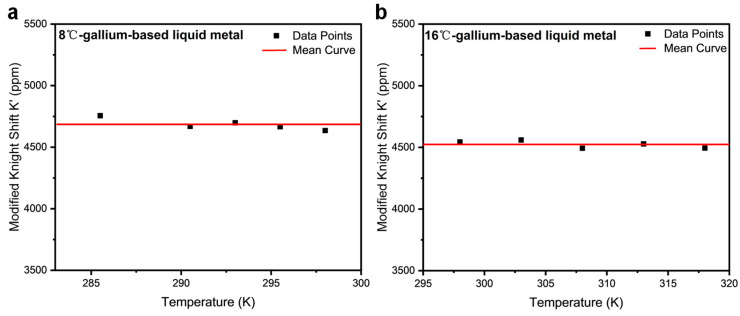
^71^Ga-modified Knight shift versus temperature (T) scatter plot for (**a**) Galinstan (285–298 K) and (**b**) EGaIn (298–318 K).

Figure 5 presents the temperature dependence of the spin-spin relaxation rate (1/T_2_) for EGaIn and Galinstan alloys. The modified Knight shift in alloys in the liquid state shows weak temperature dependence and follows the Korringa relation reasonably well, which suggests the Korringa relation could be rewritten as follows:

$$T_{2}T{K’}^{2}=28.2\;\times \;10^{-7}s\cdot k$$

The mean modified Knight shifts in EGaIn and Galinstan were calculated to be 4524 ± 45 ppm and 4685 ± 50 ppm in the liquid-state regime, respectively. In disordered liquid systems, transverse relaxation T_2_ plays a central role in restoring the validity of the Korringa relationship because it directly reflects the loss of phase coherence caused by dynamic local-field fluctuations. In such systems, strong structural and chemical disorder lead to spatial and temporal fluctuations in the hyperfine field, producing an additional broadening mechanism that shortens T_2_ but does not contribute equivalently to energy relaxation (T1). As a result, the Knight shift K, which is proportional to the static spin susceptibility, remains well defined, while 1/T1, which probes only fluctuations at the Larmor frequency, underestimates the total electronic scattering rate. This mismatch leads to an apparent breakdown of the Korringa relation when only T1 is considered. Importantly, T2 is sensitive to low-frequency and quasi-static fluctuations, including electron lifetime effects, disorder-induced inhomogeneity, and slow collective modes that are invisible to T1. Therefore, incorporating T2 restores sensitivity to the full spectrum of electronic spin fluctuations relevant to Knight shift formation. Physically, 1/T2 provides a measure of the effective electron scattering rate that governs both the static susceptibility and the dephasing of nuclear spins. Consequently, replacing T_1_ with T_2_ reconciles the experimentally observed Knight shifts with relaxation data and restores the expected Korringa scaling. This demonstrates that in disordered liquid systems, the apparent violation of the Korringa relation arises not from its fundamental breakdown, but from neglecting transverse relaxation processes that encode essential electronic dynamics.

## 4. Conclusions

In this work, we systematically investigate the nuclear magnetic resonance (NMR) properties of Ga-based liquid alloys (EGaIn and Galinstan) to resolve the apparent breakdown of the Korringa relation in disordered metallic liquids. By measuring temperature-dependent Knight shifts alongside longitudinal (T_1_) and transverse (T_2_) relaxation times, we demonstrate that deviations from classical behavior are not a fundamental theoretical failure, but rather the result of neglecting transverse spin dephasing induced by structural and electronic disorder. Our results reveal that while solid-state alloys generally follow the conventional Korringa law, the liquid phase exhibits significant discrepancies between T_1_ and T_2_. These differences reflect enhanced electron scattering, residual short-range atomic correlations, and fluctuating hyperfine fields that invalidate ideal free-electron gas assumptions. By explicitly incorporating T_2_ into a modified framework, the proportionality between the Knight shift and nuclear relaxation is quantitatively restored. This study establishes transverse relaxation as a critical parameter in liquid metal dynamics, providing a unified physical picture of electronic disorder and reinforcing NMR as a powerful local probe for designing next-generation liquid metal technologies.

## Figures and Tables

**Table 1:** Spin-lattice relaxation times (T_1_) and calculated Knight shifts (K), and spin-spin relaxation times (T_2_) and calculated Knight shifts (K’) of EGaIn measured at various temperatures.

T/K	T_1_/µs	K/ppm	FWHM/Hz	T_2_/µs	K’/ppm
258	566.5	4381.9	648.3	490.2	4710.5
263	559.6	4366.5	650.6	488.5	4673.8
268	540.4	4401.8	661.9	480.1	4670.2
273	524.9	4425.4	668.0	475.7	4648.4
278	531.3	4358.8	667.2	476.3	4603.6
283	508.3	4416.6	684.9	464.0	4622.9
298	496.3	4355.9	696.8	456.0	4544.2
303	506.7	4275.3	713.4	445.5	4559.7
308	514.3	4209.1	704.2	451.3	4493.2
313	489.1	4281.4	726.1	437.3	4528.1
318	481.6	4280.7	726.8	436.9	4494.5

**Table 2:** Spin-lattice relaxation times (T_1_) and calculated Knight shifts (K), and spin-spin relaxation times (T_2_) and calculated Knight shifts (K’) of Galinstan measured at various temperatures.

T/K	T_1_/µs	K/ppm	FWHM/Hz	T_2_/µs	K’/ppm
263	519.1	4534.0	682.8	465.8	4786.1
265.5	525.9	4483.3	704.4	451.2	4839.9
268	500.9	4572.3	705.2	450.7	4820.1
270.5	510.8	4506.8	699.8	454.1	4779.7
273	495.4	4555.4	691.6	459.4	4730.2
275.5	509.6	4470.9	718.1	442.4	4798.5
278	496.2	4510.5	712.9	445.5	4760.1
285.5	468.9	4578.6	730.9	434.5	4756.2
290.5	461.1	4577.0	717.1	443.0	4669.6
293	469.9	4514.5	732.0	433.9	4698.2
295.5	480.8	4444.2	728.3	436.1	4666.4
298	476.7	4444.5	724.8	438.24	4635.7

## Data Availability

The original contributions presented in this study are included in the article/supplementary material. Further inquiries can be directed to the corresponding author.

## Supplementary Materials

The following supporting information can be downloaded at: https://www.mdpi.com/article/10.3390/ma19091826/s1, Figure S1. ^71^Ga NMR spectra of 8℃ gallium-based liquid metal at different temperatures. Figure S2. ^71^Ga NMR spectra of 16℃ gallium-based liquid metal at different temperatures. Figure S3. The spin-lattice relaxation decay curves were derived by integrating the ^71^Ga NMR resonance signal of EGaIn at each delay time during the inversion-recovery experiments. Figure S4. The spin-lattice relaxation decay curves were derived by integrating the ^71^Ga NMR resonance signal of Galinstan at each delay time during the inversion-recovery experiments.
