# Supplementary material for: Restoration of the Korringa Relation in Disordered Liquid Systems via Transverse Relaxation (T2)
Source: Materials (Basel). 2026 Apr 29;19(9):1826. doi: 10.3390/ma19091826 (PMC13164734; doi:10.3390/ma19091826)
Supplement: Supplementary file 1 [file materials-19-01826-s001.zip › materials-4234411-supplementary.pdf]

Supplemental Information

Restoration of the Korringa Relation in Disordered Liquid Systems via Transverse Relaxation (T_2)

Yuan Zeng, Lanlan Yang, Jiejun Yao, Wei Tang and Xiaolong Liu *

School of Materials, Sun Yat-sen University, Shenzhen 518107, China

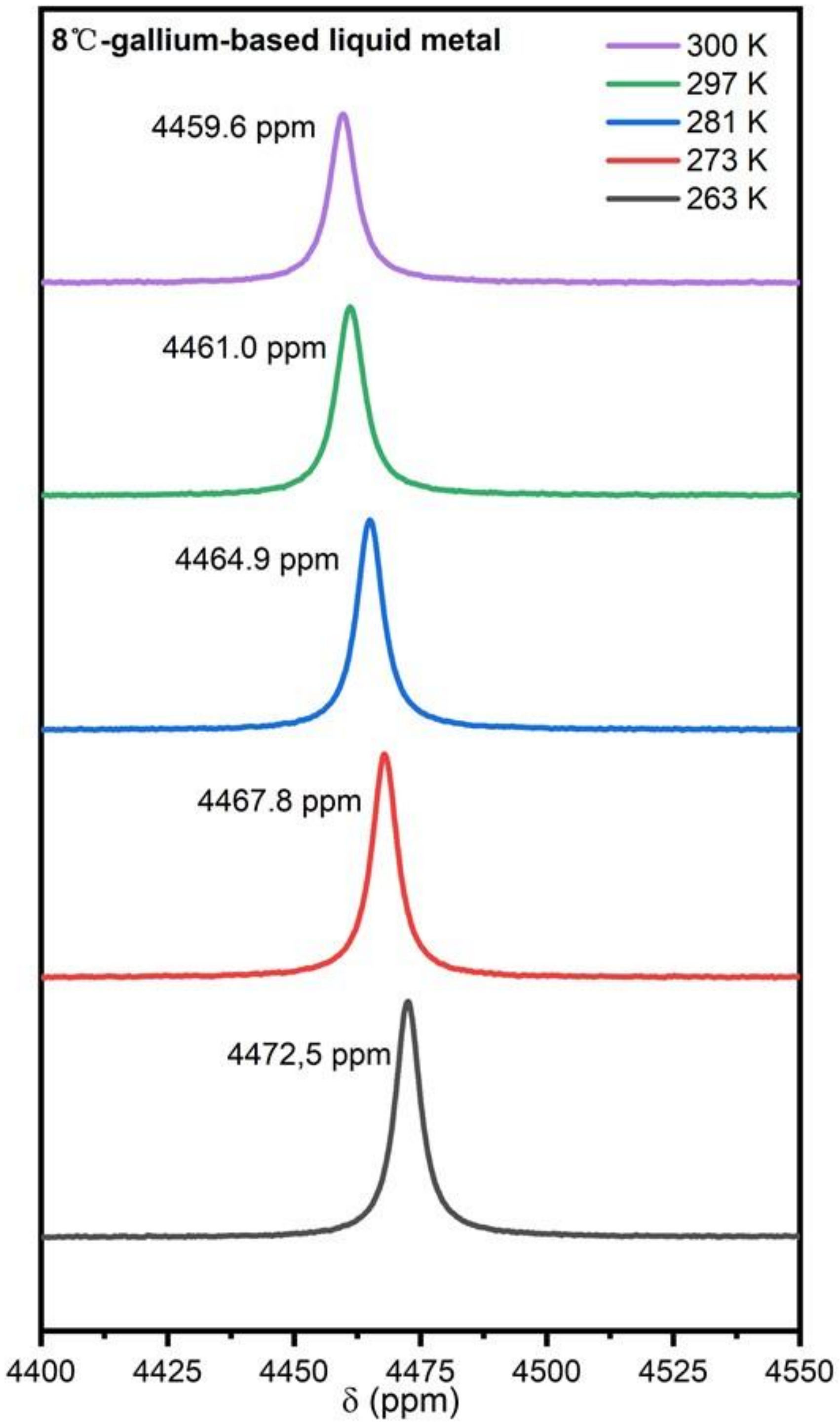

Figure S1. ^{71}Ga NMR spectra of 8°C gallium-based liquid metal at different temperatures.

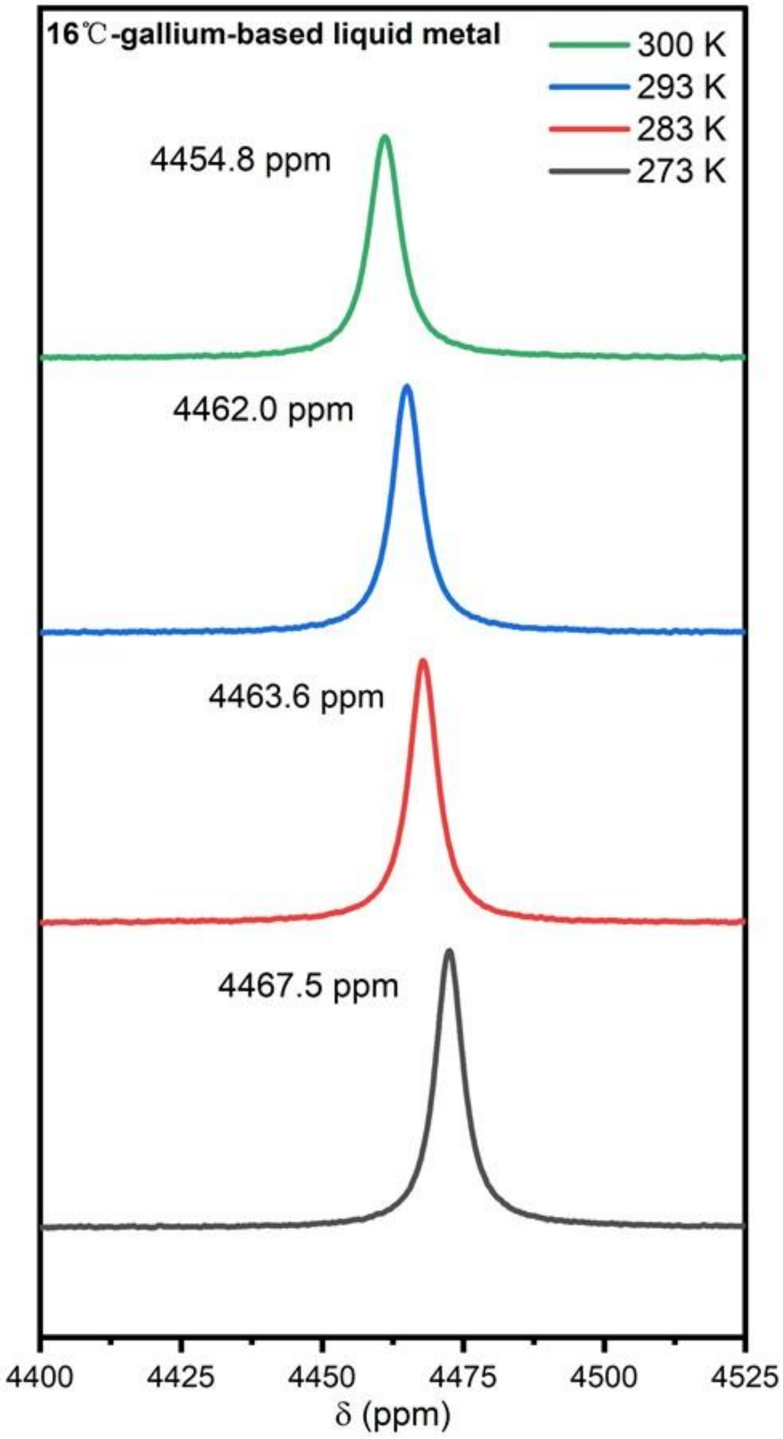

Figure S2. ^{71}Ga NMR spectra of 16°C gallium-based liquid metal at different temperatures.

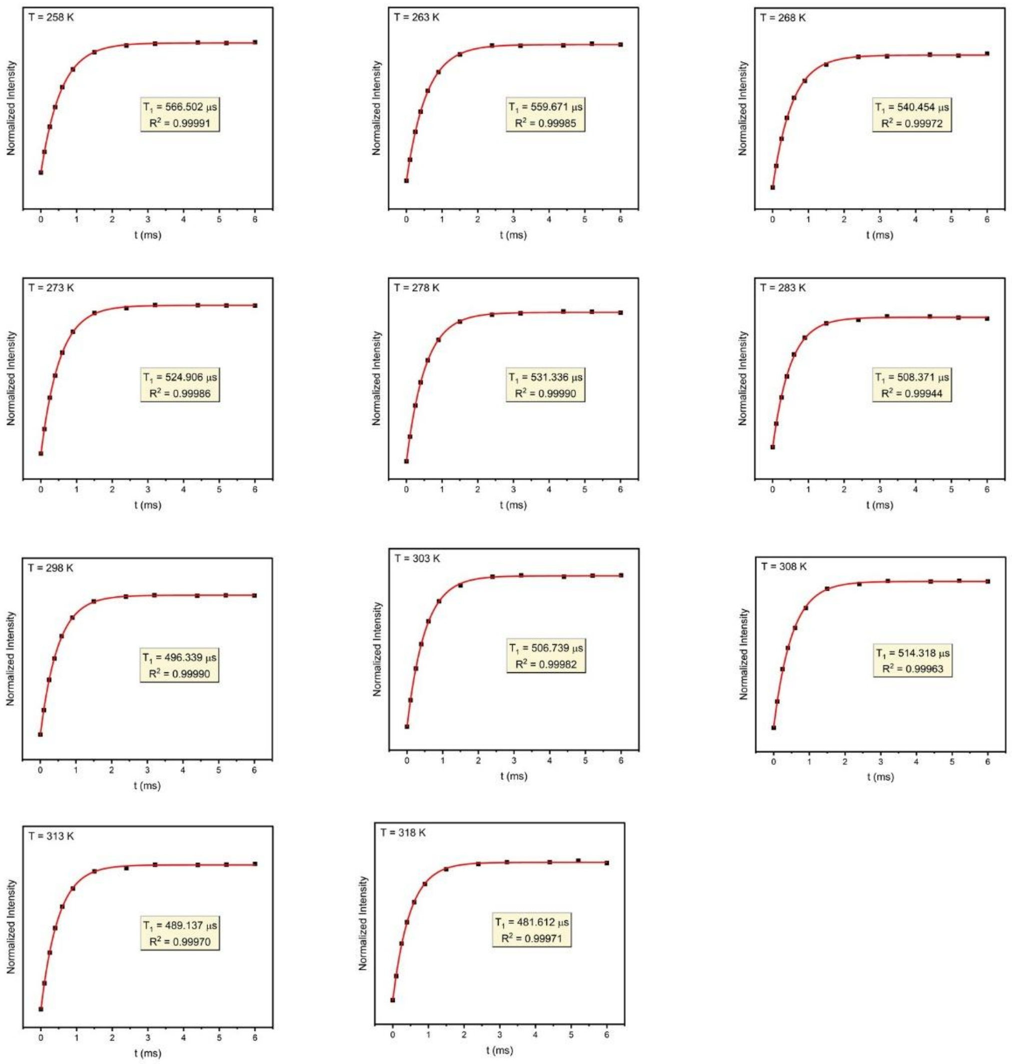

Figure S3. The spin-lattice relaxation decay curves were derived by integrating the ^{71}Ga NMR resonance signal of EGaIn at each delay time during the inversion-recovery experiments.

.

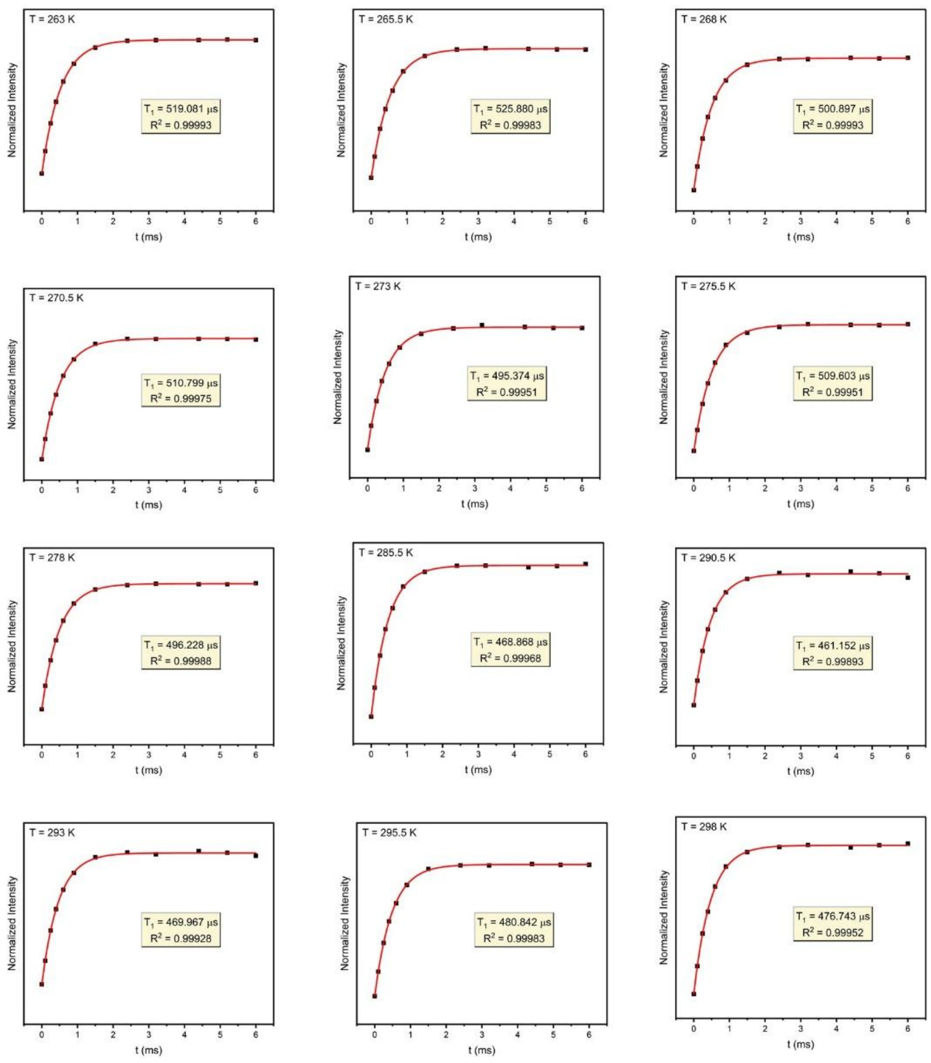

Figure S4. The spin-lattice relaxation decay curves were derived by integrating the ^{71}Ga NMR resonance signal of Galinstan at each delay time during the inversion-recovery experiments.
